# Model-driven engineering city spaces via bidirectional model transformations

**DOI:** 10.1007/s10270-020-00851-0

**Published:** 2021-02-16

**Authors:** Ennio Visconti, Christos Tsigkanos, Zhenjiang Hu, Carlo Ghezzi

**Affiliations:** 1 Technische Universität Wien, Vienna, Austria; 2 Peking University, Beijing, China; 3 Politecnico di Milano, Milano, Italy

**Keywords:** Bidirectional model transformations, Model-driven engineering, CityGML, Digital twins

## Abstract

Engineering cyber-physical systems inhabiting contemporary urban spatial environments demands software engineering facilities to support design and operation. Tools and approaches in civil engineering and architectural informatics produce artifacts that are geometrical or geographical representations describing physical spaces. The models we consider conform to the CityGML standard; although relying on international standards and accessible in machine-readable formats, such physical space descriptions often lack semantic information that can be used to support analyses. In our context, analysis as commonly understood in software engineering refers to reasoning on properties of an abstracted model—in this case a city design. We support model-based development, firstly by providing a way to derive analyzable models from CityGML descriptions, and secondly, we ensure that changes performed are propagated correctly. Essentially, a digital twin of a city is kept synchronized, in both directions, with the information from the actual city. Specifically, our formal programming technique and accompanying technical framework assure that relevant information added, or changes applied to the domain (resp. analyzable) model are reflected back in the analyzable (resp. domain) model automatically and coherently. The technique developed is rooted in the theory of bidirectional transformations, which guarantees that synchronization between models is consistent and well behaved. Produced models can bootstrap graph-theoretic, spatial or dynamic analyses. We demonstrate that bidirectional transformations can be achieved in practice on real city models.

## Introduction

Living spaces in the modern age are often complex spatial environments, characterized by an interplay of physical and computational functionalities. Such spaces host not only humans but also a wide range of computational devices—from networking components to roaming robots. As such, the overall spaces constitute complex and heterogeneous cyber-physical systems. Such is the case not only within buildings but in large urban areas as well, with the proliferation of smart functionalities being deployed and having effects across cities. As societies evolve and complexity grows, engineering complex systems supporting such spatial environments presents new challenges, where typical scenarios are dominated by information from multiple domains and the need for assurances regarding the overall systems’ behavior. In the realm of smart cities, a digital twin is a virtual model of a city—a model representation of the physical world. This conception has emerged as highly useful to reason, visualize and generally facilitate engineering of city-wide cyber-physical systems involving layered data sources of buildings, urban infrastructure, utilities, movement of people and vehicles.

Systems operating within smart environments are space-dependent, cyber-physical systems, whose development demands software engineering support facilities that span their lifecycle, from design to operation. Engineering can be enabled with model representations of their spatial environment [61]; such representations can be sourced from domain models originating in other disciplines such as civil engineering and architectural informatics. Naturally, those disciplines are dominated by their own practices, tools and domain knowledge. Design tools and approaches within them produce artifacts which are geometrical or geographical representations describing physical spaces, such as buildings or cities. Although relying on international standards and accessible in machine-readable formats, such physical space descriptions [21, 34] often lack semantic information that can be used to support their analysis as normally intended in computer science, something which hinders their consideration for software-intensive, composite cyber-physical systems.

The models we consider conform to the CityGML [18] standard which also encompasses buildings (Building Information Models—BIM [44]), widely used in practice for domain descriptions, for which numerous real-world models are becoming available [24]. The overall system inhabiting a physical space, specified by such a CityGML description, may need to satisfy certain quality attributes demanding particular kinds of reasoning—think of an architect or urban planner reasoning on the accessibility of green spaces in a city. The design may also change due to the composite system’s development cycle—for example, a transportation expert may seek to analyze an emergency evacuation scenario in the same city, by placing agents in the analyzable model and evaluating their behavior. Note that multiple such views may be derived from the same design depending on different analyses sought. We stress the fact that domain models of interest are rarely simple; they may range from small buildings to large and complex metropolitan cities.

We aim to support engineering throughout design and operation: firstly, by providing a way to derive analyzable models from spatial descriptive models (i.e., CityGML/BIM)—model-based techniques can then be readily employed on the derived models; secondly, by ensuring that changes performed on analyzable models are propagated back to source domain models in a correct manner. The analyzable model is thus a digital twin of the city: The twin should always be in sync with the events and new information coming from the actual city. Given the informational asymmetry between the two different types of models, properly synchronizing them is not trivial.

Like in all asymmetric information scenarios, in fact, the synchronization process involves decisions informed by external (and reliable) sources of information, which, if not carefully selected, might hinder the correctness of the whole process. In our specific context, for example, analyzable model changes might impact other elements when mirrored in the more concrete world of CityGML objects. When performing some operations to synchronize two models, the transformation is deemed correct if they are consistent (i.e., some equivalence relation is defined between the information contained in them) [28].

Regarding this synchronization, the typical case is naturally the forward direction, i.e., deriving models from the rich CityGML descriptions and supporting their analysis. However, more advanced use cases require supporting the backwards direction—from the abstract model back to the original CityGML description. Such cases may be found within adaptive system workflows, where some automatic procedure would changing the abstract model and subsequently reflecting changes on the domain model.

A characteristic case is system runtime, where an analyzable model may need to be kept alive, while the system is operational and populated with contextual or environmental information through monitoring. For example, a digital twin of a city can be maintained at runtime for emergency response. Subsequently, analysis performed on the model kept at runtime can provide insights or serve as input to planning processes, which may perform corrective actions in order to satisfy system requirements. Insights that analysis produces or changes that planning actions demand can be synchronized with the richer domain model. Consider planning routes of ambulances within emergency response; those may need to be, e.g., visualized upon the domain model. However, planning solutions may include inducing road closures, thus changing the model structure.

The idea is to use exactly the same spatial domain models used by practitioners to represent urban areas, buildings and city spaces and project from them some abstract and more computationally convenient representation, which can be transformed back to the original one when needed. The analyzable models we target are formally modeled topological structures—*cyber-physical spaces* [62]—enjoying well-defined semantics, where formal reasoning can be performed.

Our proposed formal programming technique assures that relevant information added, or changes applied to the domain (resp. analyzable) model are reflected back in the analyzable (resp. source domain) model automatically and coherently. The technique developed is rooted in the theory of bidirectional transformations, which guarantees that synchronization between models is consistent and well behaved. Thus, our key contribution is a technical framework based on bidirectional model transformations to support engineering of space-dependent systems. The novel bidirectional reflection facilities we provide for domain and analyzable models can be readily used to (i) derive models from spatial models occurring in practice, since CityGML models of cities are widely available, and (ii) instrument model-based development. Our framework’s concrete realization is available as open source software.^1^

To provide concrete evidence of the proposed model-based approach, we demonstrate that transformations can be achieved in practice on real city models. The present paper extends [67] in the following ways: (i) The principal technical enhancement is the concrete realization of the bidirectional transformation, (ii) the role of domain specifics and the Application Policy are further elaborated, and (iii) evaluation is expanded to illustrate edge cases of interest to practical applications, over an additional real-world city model.

The rest of the paper is structured as follows. Section 2 provides necessary background and outlines design goals and challenges. Section 3 describes the design of a bidirectional transformation between city models and analyzable models. Section 4 presents tool support, while Sect. 5 provides an assessment of the proposed approach over three case studies of real cities. Lastly, Sect. 6 gives an insight of related work in the field, and Sect. 7 concludes the paper.

## City space models and their representation

Engineering cyber-physical systems inhabiting spatial environments can be enabled with the latter’s model representations. Spatial environment descriptions are typically found in other engineering disciplines such as civil engineering, architectural informatics or architecture. We consider such spatial environment descriptions as source, *domain models*. Specifically, we adopt the ones used by practitioners to represent city-wide spaces (i.e., CityGML), since they also encompass buildings. In this section, we first briefly describe our source models, before succinctly defining the models we target, serving as the digital twin of the city. Those models are analyzable, enjoy well-defined semantics and can be used for model-based engineering purposes.

### CityGML descriptions as source models

**Fig. 1:**
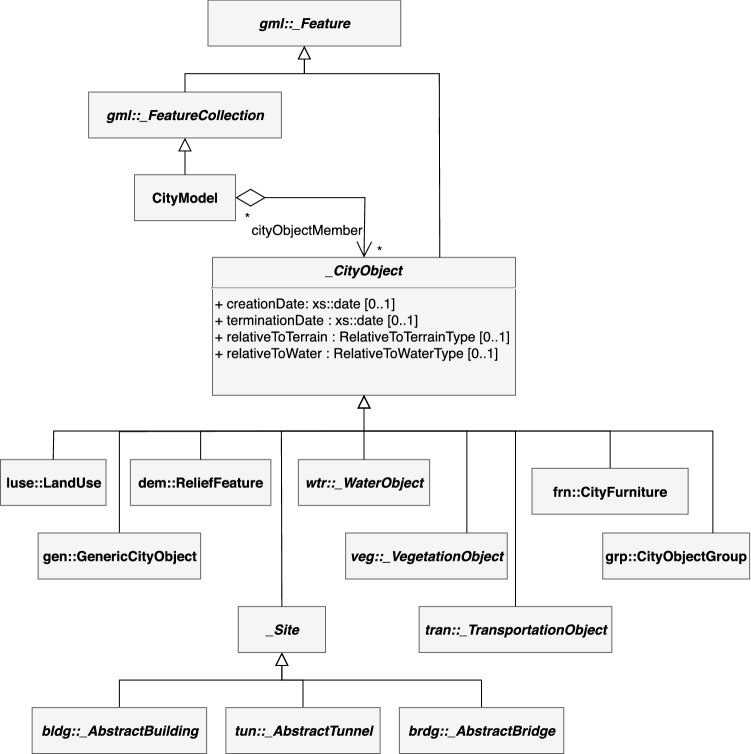
Partial view of CityGML 2.0 top level class hierarchy, adapted from [18]. Elements in italic and with a leading “_” represent abstract classes—without an explicit XML representation. Prefixes delimited by “::” when present, mark elements that belong to specific packages. Packages names comply to the CityGML naming convention recommended by the specification. Figure elements comply with the definition of thematic features as per ISO 19109

CityGML as virtual 3D city models has been widely adopted in a growing number of scenarios including urban planning, emergency management, traffic noise simulation, navigation systems, urban solar potential estimation or visual communication [14, 51]. CityGML is playing a major role, given its ability to combine both thematical and spatial representations, in progressive levels of details [18, 34]. Figure 1 illustrates elements that can be represented in CityGML. The _CityObject class plays a central role in the specification, as it defines the basic thematic properties that are extended with package-specific information. Each CityGML model has a CityModel at its root that typically contains a multitude of instances of _CityObject, through the cityObjectMember relationship. All CityGML objects extend the _Feature class of the GML language. Subclasses represent objects in some specific domains, like buildings, rivers, streets, traffic lights, etc. For a complete description of CityGML capabilities, the interested reader can refer to the specification [18].

An interesting aspect of CityGML is the flexibility it introduces, by providing a way of defining Application Domain Extensions (ADEs), in which application requirements related to the city models can be described, while the enriched model still complies to the specification [13]. ADEs are formally defined extensions, specified in XML Schema Definition or Unified Modeling Language, capable both of adding new properties to existing CityGML classes and of adding entirely new classes and data types. For example, an ADE can be a set of extra attributes and elements nested into a standard CityGML model, to extend the capability of CityGML buildings in order to fully support Building Information Modeling descriptors; this extension would affect the AbstractBuilding class of Fig. 1 by extending it with new attributes related to BIM features. This also includes adding extra elements within the ADE, which reference standard CityGML objects and describe new relationships among them. These extensions can be arbitrary, ranging from geometrical aspects like shadow orientation, to process specific, like safety escape paths. More than 40 ADEs have been developed so far, with purposes including noise propagation, energy distribution, spatial topology and time variation, among others. Despite being valuable information sources, CityGML models’ volume and domain orientation make it challenging to consider them for complex analysis and operation, requiring extensive application-specific preprocessing and postprocessing. CityGML models often include highly detailed information which may be irrelevant for certain types of analyses—for example, physical material properties of buildings are irrelevant for transportation analysis of a city. In such a case, application-specific pruning of information would need to be performed, in order to “project” the model into a transportation-analyzable view, pruning away unnecessary information.

Our technical framework has been designed under the idea of automatically migrating changes to the standard CityGML thematic features, shown in Fig. 1, and a given ADE. To the best of our knowledge, despite the many CityGML ADEs available, neither tools nor data are readily accessible for any of them as of today, and therefore, a preprocessing step is still needed in order to prepare the source information describing application-specific relationships, by referencing objects of the original city model. In the next section, to simplify the discussion, we will assume functions $\mathtt {key()}$ and $\mathtt {children()}$ are properly defined with the purposes of providing a unique identifier of the CityGML feature and retrieving a list of sub-features, respectively. Note that these functions do not exist in this form in the standard, but GML unique identification features and CityGML’s hierarchical structure can easily serve for this goal.

### Cyber-physical spaces as target models

The analyzable models that we target are topological structures termed *cyber-physical spaces* (CPSp’s [62]) whereupon formal reasoning can be performed. Such structures are the digital twin representation of a city. We opt for this generic graph-based target model because of (i) its flexibility and applicability to various types of analyses and (ii) its formal semantics, allowing for a precise definition of the correctness of a transformation. CPSp’s are graph-based representations of relations inherent in a space, which may span physical or computational barriers. This allows increased expressive power to represent complex systems and their interaction with active agents which may include devices, humans, software components or infrastructure.

Their formal semantics have been given in terms of bigraphs [42], a process meta-calculus consisting of two superimposed graphs. Such dynamic semantics are quite similar to graph transformation systems. For the formal semantics—which are not covered in this paper—the interested reader can refer to the vast body of the literature on the topic [42]. Scoped to our framework, bigraphs can be described in terms of the following components:

- A set of *labelled nodes* $\mathtt {v \in V}$ which represent the elementary objects of the environment. In the following, we will consider them as labelled with a pair $\mathtt {(identifier,type)}$ , and we assume that a $\mathtt {key(v)}$ function returning the label is properly defined. In addition, we suppose that $\mathtt {findNode(k,S)}$ is a function that returns a node *v* from the set $\mathtt {S}$ labelled with $\mathtt {k}$.
- A *place graph* is a forest, i.e., a set of rooted trees defined over nodes; this graph captures the notion of containment (nesting) of nodes. Given the structure of CityGML models, we can slightly simplify the discussion, considering that the containment relation develops from a single root representing the $\mathtt {CityModel}$ and, thus, the forest degenerates to a tree. In this perspective, we refer to $\mathtt {child(n)}$ for a node that has *n* as a parent in the containment relationship.
- A *link graph* is a hypergraph defined over the same set of nodes. Hyperedges link any number of nodes; this graph represents generic links (i.e., many-to-many relationships) among nodes. Subsequently, we suppose that a proper function, similar in principle to $\mathtt {findNode(k,S)}$, is available to find the links connecting a given node. Place and link graphs are orthogonal, and edges between nodes can cross locality boundaries.

Bigraphs allow to achieve both the level of expressiveness needed by key topological characteristics and a high level of flexibility: The place graph defines a hierarchical structure, allowing to capture locality in space of the city objects in terms of topological nesting, while the link graph can represent arbitrary connections among nodes (i.e., some other topological relation), enabling the representation of application-specific relations. Note that our choice of target model is not binding—bigraphs as used within our approach amount to general graphs with specific properties—transformation to other structures can be defined as well. However, expressing topological characteristics in terms of nesting of nodes and arbitrary relations as connections between nodes and names fits particularly well. For example, a bigraph node representing a city block may contain a number of building nodes—this is represented through nesting. A reachability relation expressing that “one can walk from a block to another via a connecting road” can be captured in the model with a link connection between two block nodes.

We refer to our target models as *analyzable*, since they enjoy well-defined semantics and can be used for automatic verification of desired properties of the overall design of the system in a formal and systematic way, as is typical in software engineering. The bigraphical representation we utilize can be integrated [31] with mainstream technologies for model-driven engineering (MDE) [15, 57], typically based on the EMOF standard [27]. In the following, we identify three major classes of analyses that may be bootstrapped by our target models.

*Graph-theoretic analyses.* By working on a topological representation of labelled vertices and edges, typical graph-theoretic analyses can be enabled on the city design, something which is not possible on the source CityGML model, which includes topology-irrelevant information. Analyses benefiting from this abstraction step to a graph may include route and network flow problems (e.g., for transportation analysis), graph coloring or partitioning (e.g., for spatial coverage or environmental analyses).

*Advanced spatial analyses.* Besides fundamental graph-based properties of the city design, more advanced requirements can be specified with spatial logics and evaluated on the analyzable models produced by our approach, referring to its topology and structure. Those can include quantitative aspects [12], which are automatically reflected on the analyzable model (such as distances in the city captured on edges, e.g., on the link graph). Furthermore, orthogonal information sources (such as sensing data) may be integrated [65]. The expressiveness that spatial logics enjoy can enable specification of properties that capture complex requirements to be evaluated on the design, while evaluation is performed with spatial model checking procedures. A characteristic case of a reachability analysis within a city will be illustrated in Sect. 5.

*Dynamic analyses.* Dynamics may be integrated in the analyzable model in order to capture ways the topology may change over time. Firstly, this may reflect design edits (e.g., possible operations that a designer performs on the city space), in the context of supporting design-time exploration of different design alternatives. Secondly (and more typically), change may refer to modeled actions by agents placed on the design aiming to analyze some complex behavior of the system [61]. In such cases, the analyzable models we target allow translation to other modeling formalisms, depending on the kind of analysis sought [62]. Typical examples of this are state-transition models supporting various forms of model checking [10, 17].

### Synchronization: design goals and challenges

In our view, model-based engineering of cyber-physical space-dependent systems should adhere to the following design principles, which underlie our design of a bidirectional transformation between the two models:

1. Interoperability with well-established domain-specific standards and data models, namely CityGML and BIM as used in practice;
2. Provision of an actionable representation of the model in a non-domain-specific language that can enable complex analysis.
3. Automatic composition of changed and unchanged parts of the model in a suitable way (i.e., well-behaved transformations), highly pertinent to both support of design activities and runtime model operations;
4. Decoupling of independent levels of reasoning (such as topological from geometrical) whenever possible, since those can be considered as being on different levels of abstraction.

We note that the biggest challenge in synchronizing a highly detailed CityGML model (originating from domain-specific tools and practices) and an analyzable model (crafted for representing high-level application-specific features in terms of topological relations) relies in keeping the consistency between the two asymmetric sources of information in both the “*forward*” direction (i.e., the abstraction process) and the “*backward*”—or “*putback*”—one (i.e., the reification process). It is particularly the *putback* direction that needs special attention, since it requires *new* information to be generated, in order to fill missing details and produce a meaningful and consistent result in terms of practitioners’ knowledge. In the following, we illustrate how the above challenges may be tackled by designing and implementing a consistent and well-behaved bidirectional transformation between source and analyzable models which, by design, properly propagates changes when either one of the models is modified.

## Bidirectional transformations of city space models

To address the problem of migrating information from one representation to another, there needs to be a clear definition of which parts of an object of the source representation have a correspondence to an object of the target representation. In other words, we have to define a correspondence between the two objects. This is typically referred to in literature as the *“consistency relation”*, among two (or more) sources of information [25]. In the following, we first succinctly describe the laws underlying our transformation and the formalization of the consistency relation. We sketch the algorithms implemented for consistency enforcement in our framework and lastly discuss some issues and limitations of the *putback* strategy in our approach.

### Consistency specification

Bidirectional transformations (BX) is a development methodology for maintaining a consistency relation between models, which can be expressed in terms of lenses [25]. More precisely, let *S* be our source city model and *V* our view (i.e., a target model), we call *lens* a pair of transformations (*get*, *put*). The *forward* transformation *get*(*S*) is used to produce a target view *V* from a source *S*, while the *putback* (or backward) transformation *put*(*S*, *V*) is used to reflect updates of the view *V* to the source *S*. In our case, we say the lens is *asymmetric* because the source model has more information than the view one. A pair of *get* and *put* should be *well-behaved*, in the sense that it satisfies the following round-tripping laws:

$$\begin{aligned} put(S, get(S))= & {} S \qquad \textsc {GetPut} \\ get(put(S, V))= & {} V \qquad \textsc {PutGet} \end{aligned}$$

The GetPut property requires that no changes in the view reflect as no changes in the source. The PutGet property requires that all changes in the view should be assimilated by the source so that the changed view can be re-computed by applying the forward transformation to the updated source.

Concerning the models we investigate, let *S* be a CityGML model and *V* a bigraph, the consistency relation between them can be formally specified in the following way. For $\forall s,s',s''$ elements and *r* relationship of *S*, and $\forall v,v',v''$ nodes of the bigraph *V*, *s* and *v* are synchronized ($s \rightleftharpoons v$) if they have the same keys ($key(s) = key(v)$) and the following conditions hold:

**A.1**: $$ instanceOf(s,\_CityObject) \wedge instanceOf(v,Node) $$;
**A.2**: ($$isContained(v,v') \leftrightarrow childOf(s,s')) \wedge s' \rightleftharpoons v' $$;
**A.3**: ($$isLinked(v,v'') \leftrightarrow holds(r,s,s'')) \wedge s'' \rightleftharpoons v'' $$.

Within the above conditions, the predicate $instanceOf$ guarantees an object is of the specified type, allowing **A.1** to define a basic level of correspondence between an element of the source and one of the view **A.2**, on the other hand, by means of $childOf$, which expresses the parent–child relationship of CityGML elements, and of $isContained$, which represents the containment relation of bigraphs and defines a mapping between two relations defined on different element types. Lastly, with **A.3**, we aim to represent an application-specific mapping: $isLinked$ represents the linking in bigraphs, while the predicate $holds$ captures both the presence of a relationship in the CityGML ADE and the fact that its application-related meaning, somehow, holds.

We may say that a source model is *place-consistent* with respect to a view model if both **A.1** and **A.2** are satisfied. Likewise, we may say that it is *link-consistent* (w.r.t. a view model) if **A.1** and **A.3** are satisfied. When a source model is place consistent and link consistent at the same time, then it is *consistent* (i.e., the models are synchronized). Place consistency has been fully formalized, and therefore, it can always be checked without ambiguity. This means that in no case, we can have, for example, a road inside a building or similar irregular cases which are not allowed by the CityGML specification. On the other hand, link consistency cannot be in principle solved unambiguously, since it is application specific. This not-completely formalized approach is not new in BX literature, since, in some cases, local correctness checks (also called black-box operations) are needed in order to achieve consistency [58].

### Consistency enforcement

The three conditions presented express, in progressive levels of consistency, that *S* and *V* are synchronized. We now present two algorithms carrying out the checks and activating the repairing procedures for guaranteeing consistency. Note that we are only describing the repairing procedures in the putback direction (i.e., the Put transformation): The description of the forward direction (i.e., the Get transformation) can be derived from it by substituting the repairing actions with some proper projection actions. It will be, in fact, automatically generated (in Sect. 4).

Algorithm 1 encompasses the first stage of the synchronization logic, where starting from the root of the city model and the outermost node of the view model, it traverses the two structures and repairs the differences by adding or removing the needed nodes at the correct position of the city model. Thus, at the end of its execution, the source model will be place consistent with respect to the view model (i.e., conditions **A.1** and **A.2** hold).
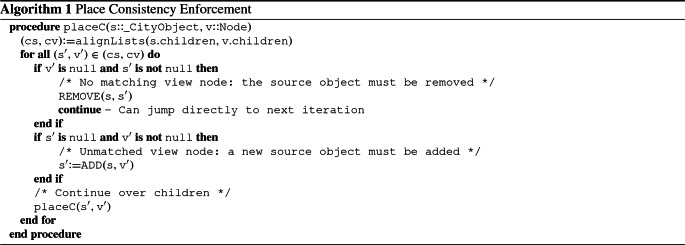


Conversely, Algorithm 2 describes the second stage of the synchronization. It starts as well from the root of the two models. However, it makes the assumption that the model is already place consistent and therefore has the only goal of repairing relationships between objects. To do so, it loops on pairs of relationships and bigraph links. If one of the two does not exist, the repairing procedure is activated. Otherwise, a further check is performed to verify that the relationship and the link reference the same elements. If this check fails, the repairing procedure is triggered. The same logic is mapped to the children nodes and continues down to the last nodes. At the end, the source model is link consistent (i.e., **A.3** holds).
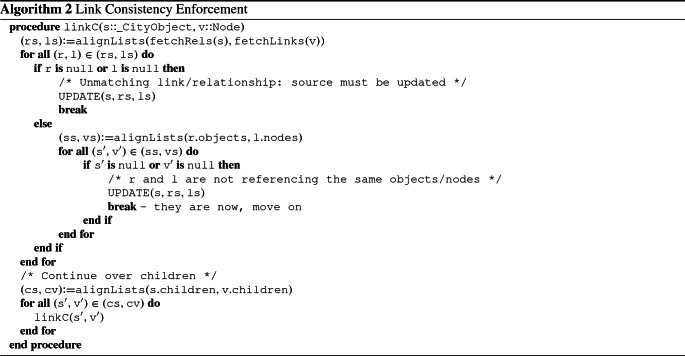


Lastly, in both Algorithms 1 and 2, procedures in uppercase represent *Application Policy* actions, which play an essential role in the transformation and are hence discussed in the next section. Illustrated functions show the functionality of the transformations in a high-level manner, and it is worth noticing that they can be executed, in the worst case, in $O(n^2 m^2)$. We defer implementation details to Sect. 4 and a more detailed complexity analysis of the algorithms presented to “Appendix B.”

### Dealing with domain specifics

Algorithms 1 and 2 are designed to satisfy the consistency conditions. However, it must be noted that albeit **A.3** specifies the consistency between CityGML relationships and graph links formally, it provides no precise information about how to implement the *holds* predicate. The reason for this choice is to keep a general approach to the transformation; in fact, the information needed to satisfy it might not even exist, since this part of the transformation is heavily application dependent. In principle, the reification strategy for new or removed objects may greatly vary depending on the purpose of the specific object and application scope and requirements. For example, removing a link that connects two buildings might mean, in one case, that the road between them is physically blocked, while in another, that moving between them is prohibited.

*Application Policy* is the component appointed for ultimately verifying that task. Since different applications are likely to require different policies, the Application Policy is an external component, interacting with our framework through clearly scoped interfaces called *actions*. Actions can access a limited set of information in order to achieve their goal, and they are required to produce an output that does not break previous assumptions.

The following actions have been defined:

- $\mathtt { ADD (s {:}{:} \_CityObject, v {:}{:} Node) {:}{:} \_CityObject }$, which is bound to generate missing objects of the source. To that extent, it has access to all the information available from the parent of the target object *s*. It can also change the representation of the parent. (This is needed in some applications, e.g., for keeping spatial-semantic coherence.)
- $\mathtt { REMOVE (s {:}{:} \_CityObject, s' {:}{:} \_CityObject)}$, which symmetrically to $\mathtt {ADD}$ has the purpose of removing extra children from the parent *s*. It has access to the same information with the same constraints.
- $\mathtt { UPDATE (s {:}{:}\_CityObject, rs {:}{:} [CityADERelati} \mathtt { on ship], ls {:}{:} [Link])}$ is the most general action, responsible for both updating ADE relationships and potentially changing the representation of the current object. The problem of correctly reflecting a set of links may be very hard to solve in general. For this reason, our framework makes two simplifying hypotheses. Firstly, we assume that a change in a relationship (or the definition of a new one) can be fully expressed in terms of separated updates to the objects corresponding to the different nodes of a link. Secondly, we assume that the information required to address this task is limited to the subgraph of nodes and links related to the current one.

To understand the generality and, thus, the complexity inherent in $\mathtt {UPDATE}$, consider a scenario in which we have two touching buildings—A and B—in the view model. A reasonable change could be, for example, to remove the *touching* relation between them and add a new one between B and C. Such an edit could be reflected in the original model in many different ways: A straightforward option could be to change the position of those objects. Another option could be to change the position of all the objects in the city to satisfy the new requirement. Our framework can currently only deal with cases of the former, since the latter changes the model so significantly that it results in a completely different one, potentially triggering an endless loop of breaking–repairing operations in other areas of the model. For an example highlighting this, the interested reader may refer to “Appendix A.” The extent to which both these interfaces and their underlying assumptions are limiting is still a matter of active investigation.

## Topocity: bidirectional transformations implementation

Having presented the classes of models of interest (Sect. 2), and the round-tripping laws guaranteeing well behavedness over which a consistency relation is defined (Sect. 3), in this section, we illustrate Topocity,^2^ the BX framework reflecting the transformations. Specifically, we present different aspects of our framework, starting from a broad picture of both internal and external components of Topocity, core points of the implementation and, finally, making some usage considerations. We adopt a functional approach to deal with transformations since they are often indeed defined in terms of functions. Moreover, this choice leads to a natural integration with the BiGUL [32, 33] library, which is a putback-based BX language developed as a Haskell domain-specific language. The primary strength of BiGUL is that, in contrast with other BX techniques, it is designed to automatically derive the *get* direction of the transformation, given the *put*. This means that developers have to implement just the backward/putback transformation from the view to the source, and the forward one is derived for free.

**Fig. 2:**
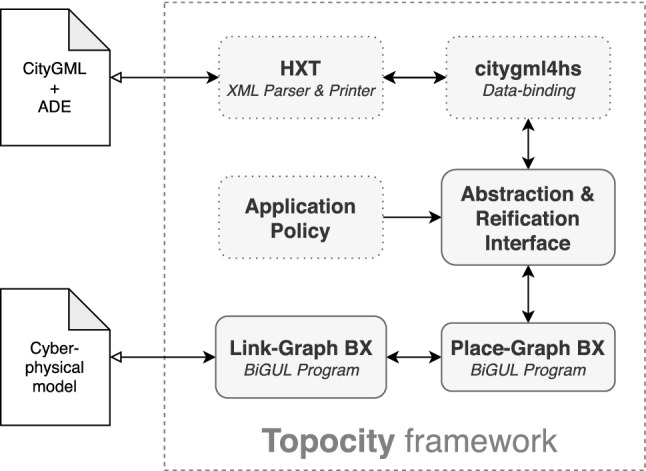
Architectural components and dataflow of Topocity. Dotted boxes represent external components

Topocity ’s main components are shown in Fig. 2; its modular design allows for external component development and integration. Naturally, functionality revolves around two models, a source CityGML description as input and a bigraph view representing the CPSp as output. Thus, we present Topocity components over the functional layers traversed in getting from one model to the other, as shown in Fig. 2.

- **HXT**. Haskell HXT^3^ is adopted for handling the XML-formatted CityGML source files in Haskell. HXT’s abstract approach allows the choice of different parsers depending on the context and couples them with the appropriate printers automatically.
- **citygml4hs**.^4^ A library providing an API for CityGML, implementing functionalities of the reference citygml4j library to Haskell. It provides a full semantic typed data structure, along with basic helper functions like the *key* method previously described. While this component is not crucial in terms of the strict requirements of the transformation, it plays an essential role in terms of non-functional requirements, since it provides an actionable representation easily exploitable by third-party software.
- **Abstraction & Reification Interface**. This component delivers a common representation of citygml4hs types. The underlying idea is that instead of building a separate BX for each object type of the source, it implements automatic algebraic transformations to generate a more abstract representation starting from the source, which can be easily converted back to the original format.
- **Place-Graph BX**. The first sweep of the synchronization process implements Algorithm 1 in BiGUL, i.e., synchronizing nodes and the place graph with the source.
- **Link-Graph BX**. The second sweep of the synchronization process makes use of BiGUL primitives to implement Algorithm 2. In contract with the previous algorithm, it synchronizes only the link graph with the source. We note that this process may be quite complex depending on the convolution degree of the link graph.
- **Application Policy**. The primary container of the framework’s API actions implements the Application Policy functionality of Sect. 3.

### Key implementation points

In the following, after basic data types definition, we elaborate on the key components of Fig. 2. We adopt a functional style for conciseness; the interested reader may refer to the online appendix for further details.

#### Data types

Recall the CityGML class hierarchy of Fig. 1: The transformation framework should address those data types. In particular, we adopt a data structure $\mathtt {\_CityObject}$ (Listing 1), with a direct correspondence between CityGML classes. The structure—part of the *citygml4hs* component of Sect. 4—is accompanied with certain helper functions providing an actionable representation that synchronization (or other external components) may utilize. For example, note that the $\mathtt {\_CityObject}$ structure not only uses a different type for each kind of city object (thus allowing implementing type-filtering selection), but also provides an Identifiable class providing a unique ID for the object (if possible) by considering the identifier hierarchy defined in the CityGML specification.
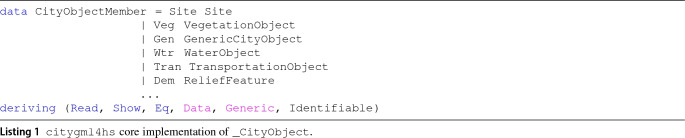


However, to reduce the complexity of the transformation, the abstraction interface transforms it into the following, more practical data structure:
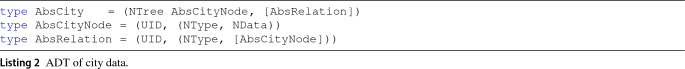


UID, NType and NData are strings representing the identifier, the object type and the internal data of each object, respectively, while NTree is just an n-ary ordered tree data structure, commonly used in Haskell. As it should be apparent from the types, the idea of this intermediate representation is to rearrange the predominant features of interest in a structure of pairs of the kind (*head*, *tail*), convenient for subsequent processing. Finally, the output data structure representing a bigraph is shown in Listing 3.
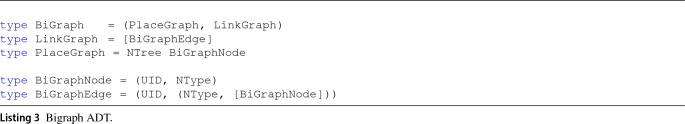


The similarities between this and the previous definition should be quite evident. This structure is equivalent to the previous, except for having dropped the NData field. That is in fact the primary difference between the two structures, since the bigraph is a *projection* that only selects a subset of the information. In principle, however, other differences could be introduced, for example, city object types and bigraph node types as different data types.

#### BiGUL programs

We have seen that the source and the view can be rearranged in a similar way without any irreversible (i.e., lossy) transformation. Our objective now becomes to migrate the information from one part to the other while keeping the correspondence as defined in Sect. 3. Concerning Condition **A.1**, we define the equivalence relation of Listing 4. The check1 function checks whether a predicate defined on two trees is true up to the first level of the subtree, which in our context entails a check of whether the keys of both the root and the children of the two trees are the same (by applying $\mathtt {(=N=)}$ on them). In this way, it is certain that when the equivalence holds, it pertains to the same object.
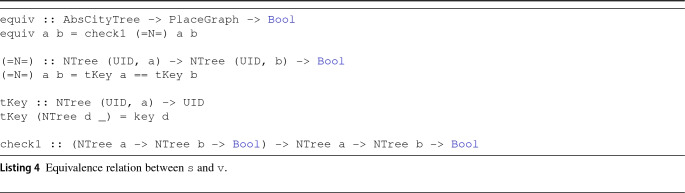


**Place-Graph BX.** The equivalence of Listing 4 is directly exploited by the *Place-Graph BX*, illustrated in Listing 5, which encodes Condition **A.2**; the implementation utilizes the Template Haskell notation used by the BiGUL language. We refrain here from presenting the details of the language; the interested reader may refer to [29] for a precise understanding of BiGUL constructs, or to [33] for proof of its correctness. Intuitively, the Case[$$\bullet $$] BiGUL operator resembles the switch/case construct of procedural languages: It takes a list^5^ of pairs of the kind (*pattern*, *operation*), where each pair describes a branch. The idea is that if the function parameters match the pattern (defined by $($$\bullet $$)), then the operation after the $${{=}{=}{>}}$$ is performed. Note the normal keyword: It is the equivalent of a procedural case having a break at the end, while adaptive corresponds to a case after which, if matched, the program continues checking against subsequent patterns in the list. Intuitively, the adaptive branch verifies that both the current element and the children have the correct ID. If this is not the case, then there might be new or missing nodes in the view, and therefore, the syncChildren function will align the two lists by exploiting Application Policy actions to create/remove nodes in the source. Conversely, the normal branch activates each time both the source tree children and the view tree children have the same id. When this happens, source types become the ones of the view, and the syncTree procedure is mapped to the children. This operation is executed recursively on all the children.
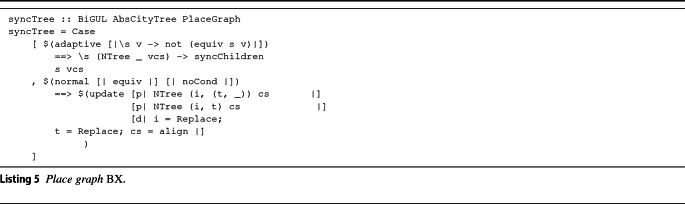


**Link-Graph BX.** After having enforced Conditions **A.1**,**A.2**, the procedure synchronizes the last part of the bigraph (i.e., the links). The procedure, illustrated in Listing 6, does this in a way not dissimilar from the previous one. The careful reader might recognize that syncGraph operates on different data types than the ones presented in Listings 2 and 3; AbsTopology and AbsHypergraph are alternative representations of [AbsRelation] and LinkGraph, respectively, indexed on nodes instead of relations/edges. The reason for this choice is the assumption that it is more convenient to loop over nodes instead of edges, since edges might not have a physical representation, but can indeed affect the way the nodes are represented as city objects. Note the syntactical similarity with the place graph BX of Listing 6; the only difference is that the procedure does not compare nodes but links defined on them and takes action when a mismatch is found. The policy action, represented as p, corresponds to the UPDATE action presented in Sect. 3. Observe that UPDATE is not activated on a single object but upon a subgraph. This graph is composed of the current node and the set of links (with corresponding nodes) defined on it and intends to provide a representation of the corresponding city objects that satisfies the set of constraints the links define. The procedure terminates with a CityGML model that is syntactically consistent with the current bigraph and semantically meaningful if a proper Application Policy is in place. If so, also Condition **A.3** holds and the two models are consistent.
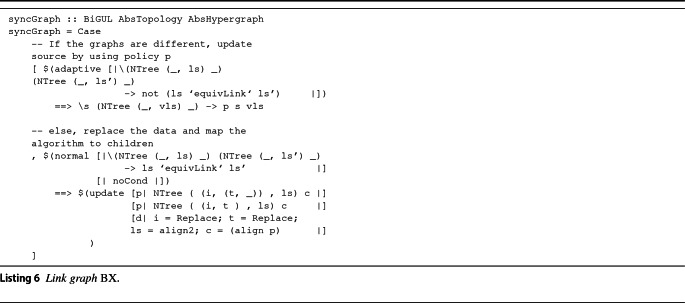


### Topocity usage

To use Topocity^6^ in practice, one is required to provide (i) a CityGML source data model and (ii) a suitable *Application Policy*. CityGML models may be obtained per-application; note that for building models, existing software (e.g., autodesk) already allow the export of drawing objects as CityGML. Repositories of CityGML city models are also available.^7^ Regarding the Application Policy, a plain one is provided by the framework by default, upon which definition of another may be bootstrapped.

Subsequently, to perform transformations, one follows these simple steps:

1. Loading of the source model (which is the pair of a CityGML and CityGML ADE description) by calling, e.g., $\mathtt {load(city.gml,ade.gml)}$.
2. Generation of a CPSp target model (i.e., perform the $get$ transformation) by calling $\mathtt {get(source)}$.
3. Generation of an updated source model (i.e., perform the putback transformation) by calling $\mathtt {put(source, view)}$.
4. Storage of the new source model by calling $\mathtt {store(filename.gml)}$.

## Evaluation

The general goal of this paper is twofold. On the one hand, we aim at giving architects, civil engineers and professionals of the field the ability to exploit the power of modern model-driven engineering techniques when designing next-generation environments. On the other hand, we aim at giving engineers the ability to develop cyber-physical systems without the hurdle of dealing with massive amounts of unnecessary information or domain specifics.

Our approach and technical framework address these problems by exploiting the BiGUL language in synchronizing CityGML data sources with application-oriented graph representations [41]. The synchronization process enforces the consistency relation defined in Sect. 3, in the way described in Sect. 4. In the following, we present three characteristic case studies that illustrate different aspects of engineering software systems inhabiting city spaces:

- While iterating within a design cycle, architects and building professionals make changes upon a design. Being able to validate decisions is crucial in assessing the quality of their design. Topocity enables deriving an analyzable model automatically from a domain-specific one. This model reflects all the changes of the original CityGML one, but in a domain where automatic analysis tools are readily available, as is typical in the model-driven engineering literature. We illustrate this by considering a reference problem from the civil engineering domain: the planning of the layout of a construction site, precisely the problem of positioning a tower crane.
- Decision making with constrained resources and in limited time is typical at runtime settings, where information is monitored from the environment. However, in many applications such decisions are non-trivial and require proper evaluation of contextual information. Analyzing a model of the environment becomes key to provide critical insights or to support planning processes. Topocity can reflect updates to an analyzable model of the CityGML source so that they can be combined with other data sources. We illustrate this characteristic case by examining an emergency response scenario in a large city area.
- Whether at runtime or design time, other non-functional concerns may arise; the most obvious one is the relation between model size and transformation performance. We investigate this issue by showcasing different cases at the extremes of the spectrum: Given a model of the city of Vienna, we point out the efficiency of the transformation. To vary model size in a controllable manner, we control the density of bigraph links over the same model.

The cases we consider are reference problems for our evaluation purposes: Each of them is representative of how bidirectional transformations can play a primary role in the engineering of systems inhabiting city spaces, enabling separation of concerns between different domains. We stress that in the three evaluation cases considered, models come from real and public CityGML data sources of, respectively, a district of Remscheid (North Rhine-Westphalia, Germany), Flat Iron Street in New York (NY, USA) and the city of Vienna (Austria). We conclude with a discussion.

### Facilitating system design: tower crane positioning

Proper optimization of construction site layout is key to efficient construction activities. Before construction starts, site layout planning provides the necessary equipment and temporary facilities for the construction process, including allocation and dimensioning of elements like tower cranes, containers or storage areas. Decisions taken during this planning phase have direct impact on cost development and occupational safety on site during construction. Positioning of tower cranes is an important exemplar [2, 30]. Recent literature has provided techniques to automate the solution of this task, where two critical issues have been identified: (i) the lack of a simple but formal language capable of expressing rules, standards and best practices to check a building model [53] and (ii) the absence of tools able to perform this kind of operations by exploiting BIM/GIS descriptions like CityGML models, so that meaningful solutions can be found before implementation takes place [30]. In the following, we demonstrate how a flexible solution can be designed in which our framework plays a central role.

**Fig. 3:**
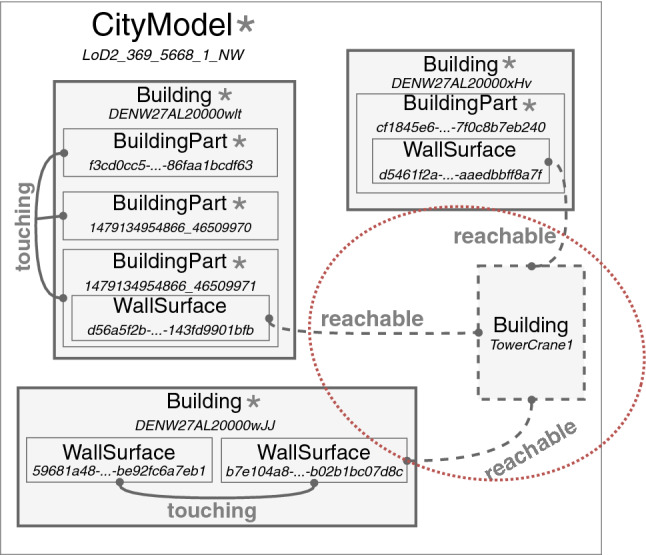
Fragment of the view model derived from the CityGML description of a district in Remscheid. Nodes are (ID,Type) pairs as they appear in the real CityGML model. Presence of other—not shown—elements of the model is indicated by *

We consider an hypothetical construction site to be placed in a district of the city of Remscheid, North Rhine-Westphalia, Germany. For the real CityGML models, we rely on North Rhine-Westphalia open data [71]—the linking structure related to tower crane positioning is designed ad hoc, since this step could be easily generalized and reproduced by modern user-guided CAD software [49]. Figure 3 shows the most relevant part of the model generated by our framework; an extra object and extra links are shown, corresponding to the changes made to the cyber-physical space in order to elicit the topological requirements for the new tower crane. Advanced analysis and model processing to generate such changes can take into account topological information in the analyzable model, such as proximity of construction site elements or complex relationships in the space layout, positioning the crane in a manner that satisfies some occupational safety or optimal placement requirements. As we are concerned with model transformations only, we consider such reasoning facilities as out of scope for this paper.

Once the target model is updated reflecting some reasoning (e.g., identifying the optimal position of the crane), changes have to be reflected back to the original model. To this end, Topocity takes care of identifying changed objects and prompts the Application Policy to provide the 3D shape of the tower crane and spatial coordinates. For our case study, this was a fixed position, but a policy can specify arbitrary alternatives, from random to user-defined positioning, depending on the kind of links defined. Once those are given, Topocity identifies the place in the original source hierarchy to arrange the new objects and reifies the model back again to the CityGML description.

**Fig. 4:**
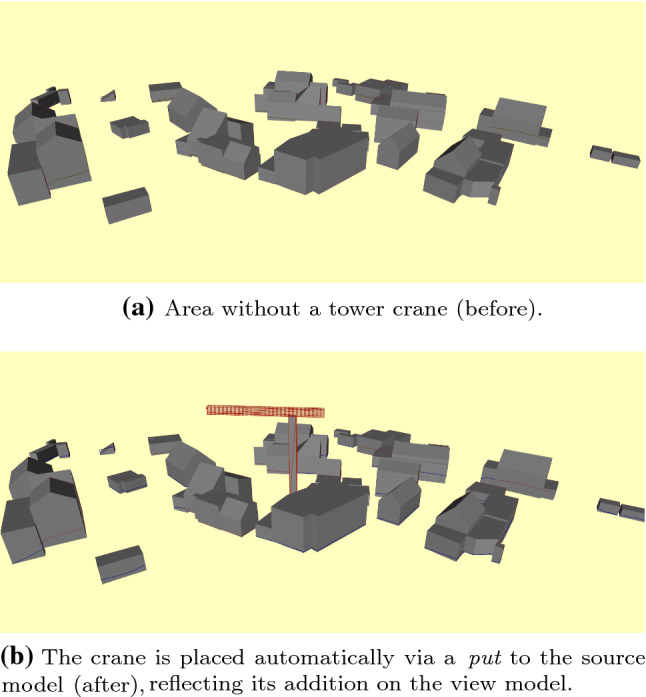
Placement of a crane entity on the derived, analyzable model (Fig. 3) entails its automatic reflection on the source city model (Fig. 4a), resulting in Fig. 4b

Figure 4 shows a fragment of the original model and the final result as visualized CityGML descriptions. Note how certain reachability links between edges of three buildings are additionally defined, supposing these are buildings of interest for the construction site (Fig. 3).

### Facilitating system operation: emergency response

Technology adoption for fast emergency response in urban environments is gaining increasing attention: Technological advances may in fact provide new human–computer interaction capabilities, allowing for effective real-time response. Consider the classical setting [37] where a disaster scenario is replicated in the Flatiron Building area of New York [43], with several relief entities (e.g., rescue teams, ambulances or Unmanned aerial vehicles—UAVs) dispatched throughout the area to locate and rescue victims [20, 62].

The agents have initial knowledge of the environment, given by the original model of the city. However, in such a scenario, we expect the model to be updated regularly, as soon as new information is acquired by monitoring processes. Agents must dynamically adjust search operations and rescue priorities through some criteria such as the likelihood of finding victims in an area or current disaster propagation. In order to perform such tasks, which largely amount to *planning* and *surveillance* [22], an actionable representation of the city can be a hypergraph in which nodes represent city objects, while links represent safe connections between multiple nodes. This typically occurs within a Monitor-Analyze-Plan-Execute loop, as this is an instance of a self-adaptive system. Agents monitor the area and update the model with the information they collect about safety of streets and buildings, while others escort civilians from the disaster area to hospitals. Path planning takes place based on analyzed monitored information upon the model, with the purpose of, e.g., maximizing the number of victims rescued. We are solely concerned with synchronization of the models—as such, analysis, planning and monitoring are therefore out of the scope of this paper. We note that analysis can be performed with spatial model checking—specification of the desired property would occur within a logic as spatial properties [62].

**Fig. 5:**
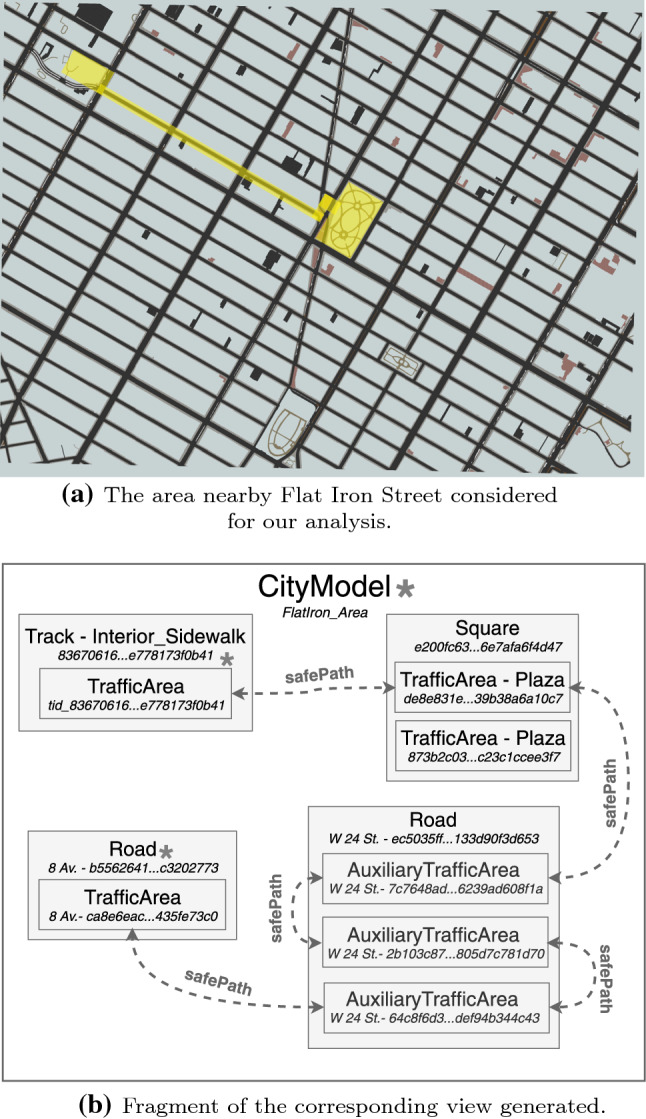
Runtime safe path analysis models. The source (**a**) is transformed into the analyzable model (**b**). The highlighted area in (a) represents the safe path illustrated in (**b**). Nodes are ID-Type pairs as they appear in the available CityGML model of New York; the presence of other elements in parts of the model (not shown) is indicated by *

In our approach, we define and extract a CityGML ADE from the city model and populate it with real-time information, with the goal of making the *safe distance* relation between city objects explicit. Topocity provides the hypergraph exploited by the agents, which is updated at runtime as the monitoring process generates new information. Figure 5 shows the aerial view of the Flat Iron Street area of New York as described by the CityGML model (5a) and the corresponding analyzable view (5b). A viable safe path for the city area is shown, both in the original model and in the analyzable one.

### Edge cases analysis

**Fig. 6:**
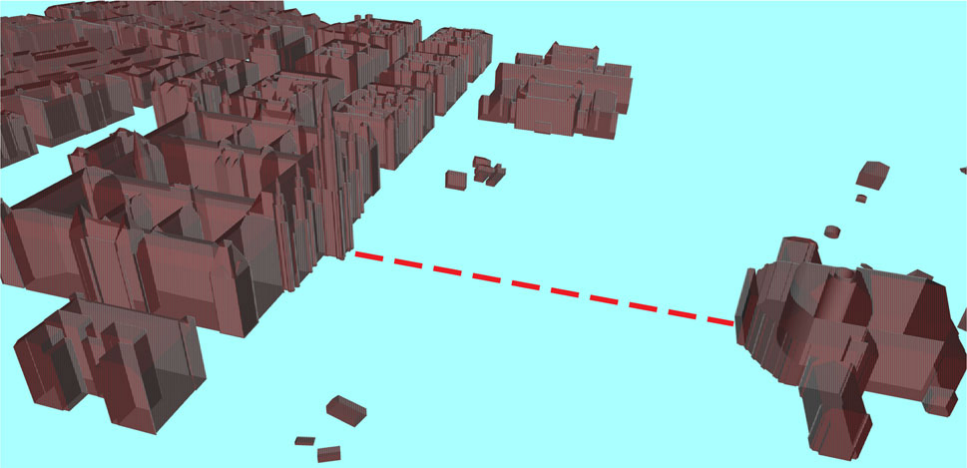
An hypothetical reachability relation between Rathaus and Burgtheater in Vienna (Austria)

Different applications—even if related to the same geographic space—might likely need to deal with very different models. This diversity can appear as well among CityGML models, where Application Domain Extensions might significantly affect both the size and the purpose of the model. Imagine, for example, a scenario where engineers have to take decisions based on the distance of two objects in a city (shown in Fig. 6).

More precisely, let *k* be a positive number representing the maximum distance they are willing to consider. Two objects are *k-reachable* when the minimum distance between them is smaller than *k*. We can say that an object is *0-reachable* from another if the two are touching. At the same time, we can say that every two objects in the city are $\infty$-*reachable*. Depending on the value of *k*, the size of the analyzable models can grow significantly: Higher values mean we are more tolerant in the definition of reachability, and therefore, more relationships will be encoded in the CityGML ADE, resulting in more convoluted link graphs. We investigate the effect of this variation, over the central area of the city of Vienna, for several values of *k*. The analyzable model generated contains 1120 nodes and up to 448 thousand links.

Table 1 reports a summary of the performance of the forward transformation executed on the model of the central area of the city of Vienna. The experiments were performed on a machine equipped with an Intel(R) Xeon(R) CPU E7-8880 v3 @ 2.30GHz CPU and 961 GB of RAM (to represent unlimited memory) on Amazon AWS.

The performance of the transformation on this non-trivial model provides a twofold insight for working with large-scale systems. The first is that the XML parsing stage primarily affects the space impact of the program. This means that working with a chunked model or pre-parsed data (e.g., by obtaining data from a database) will dramatically reduce, if not remove, the overhead of this stage. The second is that times quickly degrade with the increase in the number of relationships to analyze. While this conforms with the behavior of algorithms of Sect. 3 and their theoretical time complexity (see “Appendix B”), it is nevertheless clear that more efficient data structures and algorithms, paired with parallel computation, might significantly mitigate this effect. Lastly, it must be noted that we have been considering sizes far bigger that the typical sizes supported by commercial tools: It is very likely that instead of having a dense network of relationships connecting all objects of the city, more clever source representation will be in place in practical scenarios. Naturally, processing entire cities in a single model is not the norm in CityGML workflows, which typically consider fractions at a time. Our experiment’s purpose, however, was to systematically investigate consideration of large models, through varying values of *k*.

**Table 1:** Comparison of results on the model for the central city of Vienna. The source is represented as an uncompressed XML file, while the view is an uncompressed binary dump of the target data structure. Source models are obtained from [56]

100%	Source	Parsing		Transformation		View
	Size (MB)	Time (s)	RAM (MB)	Time (s)	RAM (MB)	Size (MB)
(*no* *reach*)	42	24	810	1	130	2.7
$$0-reach $$	43	25	810	259	320	3.3
$$10-reach $$	44	25	810	643	320	4
$$20-reach $$	45	26	810	1166	350	4.8

### Discussion

The three exemplar cases presented are different, as (i) they target different models and different levels of detail within CityGML and (ii) they showcase uses of the framework for both systems’ design and operation and (iii) they are evaluated on models of different density, spanning from a tree structure to a dense graph over a non-trivial set of nodes. Hence, we believe they show the potential of our approach. By using our framework, bidirectional model transformations upon real spatial descriptions can be performed, keeping analyzable models and CityGML descriptions synchronized. However, from our experience within model transformations of CityGML descriptions and considering the perspective of practitioners aiming to use our model-based engineering approach, interfaces and tooling integration might significantly support the design cycle.

A significant flexibility constraint has been briefly presented in Sect. 3.3. As anticipated there, links can be a very powerful medium for expressing arbitrarily complex configurations: In some convoluted scenarios, a putback to the original model may not be feasible or even worse; it may result in changes affecting a vast number of features, essentially resulting in a different model. We believe our solution addresses a relatively general set of meaningful applications, but further research on application scenarios may result in more precise understanding of practical limitations. Moreover, a considerable problem in making our framework an effective tool for practical use is the absence of any public ADE data or generation tool. Nonetheless, we believe this limitation may soon be overcome, thanks to the growing interest in the CityGML standard by domain experts [13].

An important aspect in BX design is the level of automation desired—ideally, one would expect to be able to choose an Application Policy that meets certain needs, plug it in the Topocity framework and use the combinations of these programs with no extra effort, regardless of the application context. However, our experience shows that some very complex CityGML features containing highly varying objects, still need some minimal custom bridging code to build the transformation. Tackling this problem in a generic manner requires extending the approach, something we identify as future work.

It is worth mentioning that [53] already solves the tower crane problem of Sect. 5-A by developing a plugin for Autodesk Revit—an established tool in building and urban design. However, as pointed out by the authors, only a small set of pre-defined simple rules are allowed, implemented ad hoc for this purpose. In addition, [30] shows that GIS-BIM models (like CityGML) have enough information for treating the problem in terms of geometrical and topological analysis. Our approach, on the contrary, is general enough to allow for complex rules and user-defined customization if a proper Application Policy is set in place.

The first two cases considered for our evaluation purposes are model problems obtained from domain-specific literature, highlighting the use of bidirectional transformations within our framework for model-based engineering of space-dependent systems. We believe that the strength of our approach is twofold: Firstly, adaptability is exhibited, since integrating disparate application-related sources of information still result in the same analyzable model; secondly, providing an automatic way to obtain an abstract model where verification can be performed can lead to the development of more sophisticated analysis-based workflows.

Finally, within the general context of engineering systems inhabiting city spaces, we illustrated two characteristic use cases where the approach we advocate can be beneficial. Those highlight a model-driven adaptive systems engineering view. At design time, development is grounded on modeling activities, including processing and analysis of whether the system inhabiting the city space satisfies its design goals. However, after it has been designed and deployed, goal satisfaction may depend on environmental information that arise only in operation. Such information may need to be integrated to the city model to enable processing and analysis, but in this case, this has to be performed at runtime, where the model is populated as information arrives. Thus, both for design time and runtime cases, analysis and processing cannot be performed upon the CityGML domain models, but on the analyzable models that our framework derives. Keeping domain models in sync with derived analyzable models is crucial.

## Related work

We have presented a novel technical framework to engineering bidirectional model transformations of city models, offering assurances on correct and well-behaved transformations. Consequently, we classify related work into three categories. First, we discuss the state of the art in model-based analysis of physical spaces, positioning our work. Then, we review transformation techniques and theoretical foundations on consistency. Lastly, we discuss related engineering approaches from the domain of analyzable models (i.e., cyber-physical systems that build upon spatial representations).

Interest on model-based analysis of cities has been consistently growing in recent years. The adoption of CityGML for building modeling purposes has been studied extensively lately [45, 59, 72], and the integration of classical BIM features has been a leading design goal [54] in defining CityGML 3.0, to be soon released [36]. In addition, city-based analysis is being developed in all kinds of application scenarios; most notably, recent efforts have been on traffic noise analysis [35], photovoltaic potentiality analysis [7], urban emission measurements analysis [5] and ubiquitous robot networks management [60]. Official city datasets are increasing, with recent public effort from Turkey [9], Singapore [55] and Germany [71] among all.

Bidirectional transformations (BX) have been an active area of research for many years now, with a growing research community and a dedicated conference (“BX,” since 2011). Transformations address the problem of defining consistency between models, historically originating from the view-update problem in database research [11, 48]. An introduction to the topic of BX can be found in [4], whereas a very recent comprehensive overview of the field and techniques for assessing their performance is available in [8]. Several different approaches have been studied for dealing with bidirectional transformations. BiGUL is a formally verified putback-based bidirectional programming language [32, 33] based on lenses; Symmetric Edit Lenses [68] could be used alternatively, although no practical tool is available to date. The most popular alternative approach to lenses is the relational one, on which QVT and its relation language (QVT-R) are predominant [26, 39, 40]. Another major alternative approach is the Triple Graph Grammar (TGG) [28, 52], usable by the BXtend [16] tool. The flexibility (and hence the limitations) of our reflection facilities is greatly related to the theoretical issues with the propagation of effects. Arguably the first work to clarify these issues is [58]; since then works address effectful bidirectional transformations [46] and monadic lenses [3, 46] and have been dealing with side effects in general. Development of practical tools exploiting these theoretical results is object of active research by the community.

Different forms of graphs as formal models of static representations of buildings or cities have been proposed in diverse fields such as architectural informatics [38] or computer graphics [70], with different objectives. Several approaches target case-based reasoning [1] in the architectural domain. However, actionable and analyzable models are necessary for advanced design and operation of overall space-dependent systems [61]. In [38], a topology of spatial configurations is extracted from building information models as well as handwritten architectural sketches [6] and represented as graphs. Within the Internet of things, analyzable models are extracted from trajectories and reasoned upon with a spatial logic [65]. Focusing on security reasoning while aiming at early design phases, Porter et al. [47] propose a method and heuristics to discover security threats on building specifications via simulation. Analyses such as similarity checking are performed based on graph matching techniques [19]. Forms of graphs representing topology of space are highly useful. To this end, our target analyzable models are graph-based and readily analyzable with a variety of approaches [64]. The notion of a cyber-physical space refers to a composite model able to capture complex relations of human, cyber and physical entities, which may span physical or computational barriers. Such a model may be obtained from a physical model and enriched with formally specified dynamics capturing possible ways it can change [63]; spatiotemporal model checking of evolving cyber-physical spaces can then be considered [62]. We note that different model projections corresponding to requirements may be derived (and synchronized) on the specification level automatically, achieving cone-of-influence reduction on analysis [66].

## Conclusions and future work

Motivated by model-based design and operation of space-dependent systems, we presented a technical framework enabling synchronizations between city spatial domain models and graph-based analyzable models. Synchronizations produced are automatically derived, correct and well behaved. The models we considered are based on CityGML, widely used by practitioners to represent city or building spaces. The novel bidirectional reflection facilities we provided can be readily used to (i) derive models from real CityGML models occurring in practice and (ii) instrument modeling and analysis facilities for cyber-physical systems. Their realization in the form of an accompanying artifact is available as open source software.

Considering the perspective of practitioners aiming to utilize model-driven synchronization facilities, we identify several research directions that could be pursued in the future. Interfaces and toolchain integration would go a long way in supporting the design cycle. This goes hand in hand with tackling practical issues of CityGML, such as public ADE data or generation facilities, to ensure effective tooling for practical usage. The general class of synchronization problems we addressed must be clarified: Our framework’s main hypothesis is the unchangeability of the consistency relation between the source and the view; an alteration to the latter may require massive rewrites of the core of our framework. Regarding theoretical aspects, we aim to investigate pluggable custom application policies and support arbitrary CityGML features. Lastly, an interesting research direction is the generalization of synchronization to a many-to-one model; being able to support multi-model sources might enable support of a wider range of applications dealing with wide area services (such as transportation networks, telecommunications, etc.).

## Figures and Tables

**Fig. 7:**
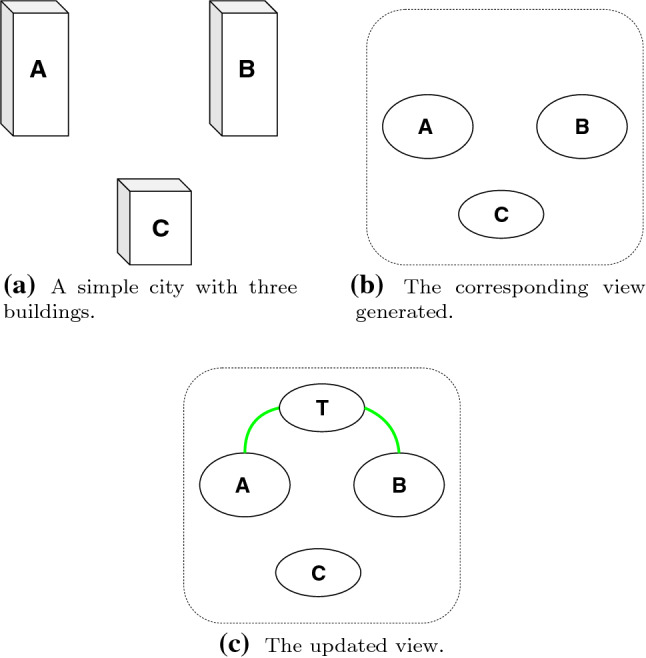
A typical problematic case. There are three buildings in our city (**a**), and we start to analyze them without any extra relationships - no links in view (**b**). Then, we decide we want to connect A and B with a tunnel T (**c**)

**Fig. 8:**
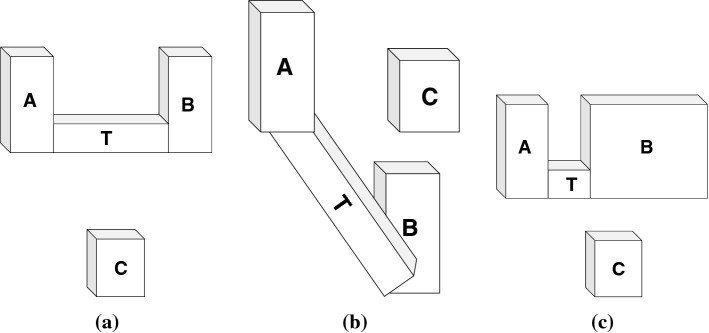
Despite all these solutions being formally correct, only solution (**a**) is reasonable, since it does not require to move other buildings or to change their shapes

## Application policy example

To get a concrete understanding of the issue, imagine a setting like the one in Fig. 7: let’s say we want to add a tunnel that connects buildings A and B. Figure 7c shows the bigraph expressing this requirement. To reflect the changes back to the city model, let’s say we start iterate the putback procedure starting from the building A; there are clearly many alternative solutions; some of them are shown in Fig. 8.

By looking at the picture 8, one can see that there could be theoretically multiple (actually infinite) solutions to the problem. Our UPDATE action would only allow solution (a), because solutions (b) and (c) would require to change the shape or the position of nodes different from the current one, which are two illegal operations for our Application Policy.

In the most degenerate case, a solution may even result in a completely different configuration of the city space, where for example the position of every object in the city is changed in order to satisfy a requirement. In such cases, it would become meaningless to synchronize the two models because, as it is even harder to say that we are talking about the same model as the beginning. It must be noted that transformations like cases (b) and (c) might indeed be useful in some cases. However, we did not find, to date, any suitable case scenarios for them and the limiting factor of this assumption is still a matter of active investigation.

## Complexity analysis

Let $\mathtt {objects}(S)$, $\mathtt {relationships}(S)$ denote, respectively, the set of objects and the set of relationships of the source. Conversely, let $\mathtt {nodes}(V)$ and $\mathtt {links}(V)$ denote the sets of nodes and links of the view, respectively. We denote by $|\cdot |$ the cardinality measure of a set. We also call a *trivial policy* any Application Policy of the kind described in Sect. 3, running in *O*(1) (imagine for example constant policies, which reify nodes as objects of a pre-defined fixed shape at a pre-defined fixed position).

### Lemma 1

Place consistency of a trivial-policy *put* transformation between a CityGML model *S* and a bigraph *V* can be obtained in $O(n^2)$, with $n = max\{|\mathtt {objects}(S)|,|\mathtt {nodes}(V)|\}$.

### Proof

By definition, the transformation from *V* to *S* is place-consistent if **A.1** and **A.2** hold, which are the postconditions of Algorithm 1. First, assume objects and nodes already have references to their children readily available in their data structure (for, e.g., like in Listing 2). The starting operation performed is $\mathtt {alignLists(\cdot )}$ which—since it can be seen as a form of string alignment—can be executed, in the worst case scenario, in $O(n^2)$ [23, 50, 69]. The algorithm, after aligning the lists, loops over the pairs of source objects and view nodes. In the worst case, the source and the view have no elements in common, which means the loop runs $|\mathtt {objects}(S)| + |\mathtt {nodes}(V)|$ times, which is less than $O(2n) \approx O(n)$. Supposing the source and the view are completely different, any iteration of the loop might trigger our Application Policy, which returns a result in *O*(1). Lastly, this behavior is mapped to every element of the two trees. However, this does not change the complexity, since at previous steps we had two trees having all the nodes as direct children, which represents the worst case. (The most computational-intensive operation is performed on all the elements and nodes of the two trees.) In conclusion, the time to execute the algorithm is $O(n^2) + O(n) * O(1) = O(n^2)$.$\square$

### Lemma 2

Link consistency of a trivial-policy *put* transformation between a CityGML model *S* and a bigraph *V* can be obtained in $O(n^2 m^2)$, with $n = max\{|\mathtt {objects}(S)|,|\mathtt {nodes}(V)|\}$ and $m = max\{|\mathtt {relationships}(S)|,|\mathtt {links}(V)|\}$.

### Proof

Similar to Lemma 1, we are considering a policy of complexity *O*(1), and we are enforcing **A.3** as a postcondition of Algorithm 2, which has **A.1** and **A.2** as preconditions. First, $\mathtt {fetchRels(\cdot )}$ and $\mathtt {fetchLinks(\cdot )}$ do, symmetrically, the same operations on the two structures, i.e., they create an auxiliary data structure that indexes the relationships/ links in terms of the objects/nodes they relate to. In the worst case, where relationships and links refer all the objects and nodes, respectively, this operation can take at most $O(|\mathtt {objects}(S)|~|\mathtt {relationships}(S)|)$ for $\mathtt {fetchRels(\cdot )}$ and $O(|\mathtt {nodes}(V)|~|\mathtt {links}(V)|)$ for $\mathtt {fetchLinks(\cdot )}$, therefore $\approx O(nm)$. Now, with arguments similar to Lemma 1, $\mathtt {alignLists(\cdot )}$ can be executed in $O(m^2)$ [23, 50, 69] and the outer for loop iterates at most $|\mathtt {relationships}(S)| + |\mathtt {links}(V)|$ times, i.e., *O*(*m*). Now, the internal $\mathtt {alignLists(\cdot )}$ could still take up to $O(n^2)$, in the case a relation/link connects all the objects/nodes of the respective structure. This whole procedure is executed once for each object or node (note that they are exactly *n*, since **A.1** holds). At the end, we have $O(n) * [ O(nm) + (O(m) * O(n^2) * O(1)) ] \approx O(n^2 m^2)$. $\square$

### Proposition 1

A CityGML model *S* and a bigraph *V* can be made consistent by a trivial policy in at most $O(n^2 m^2)$, with $n = max\{|\mathtt {objects}(S)|,|\mathtt {nodes}(V)|\}$ and $m = max \{|\mathtt {relationships}(S)|,|\mathtt {links}(V)|\}$.

### Proof

By definition, *S* and *V* are consistent if they are place consistent and link consistent, which, as proved in Lemmas 1 and 2, can be enforced in $O(n^2) + O(n^2 m^2) = O(n^2 m^2)$.$\square$

## Footnotes

1 Topocity is available at https://topo.city.

2 Available at https://topo.city or https://www.github.com/ennioVisco/topocity.

3 HXT—https://intern.fh-wedel.de/~si/HXmlToolbox/.

4 https://www.github.com/ennioVisco/citygml4hs is maintained by the authors as open source software.

5 Lists are enclosed in [ ] in Haskell.

6 For installation and example usage, the interested reader is referred to https://www.topo.city.

7 Examples include TU Delft models of Dutch cities https://3d.bk.tudelft.nl/opendata/3dfier/; the TU Munchen model of New York https://www.lrg.tum.de/gis/projekte/new-york-city-3d/; North Rhine-Westphalia models https://www.opengeodata.nrw.de/produkte/geobasis/3d-gm/. An extensive list is available at [24].
